# Supplementary material for: Mechanisms governing the pioneering and redistribution capabilities of the non-classical pioneer PU.1
Source: Nat Commun. 2020 Jan 21;11:402. doi: 10.1038/s41467-019-13960-2 (PMC6972792; doi:10.1038/s41467-019-13960-2)
Supplement: Supplementary file 7 — Source data [file 41467_2019_13960_MOESM7_ESM.zip › Source_Data/FigureS3/FigureS3J_MotifScanOutput/homerResults/motif24.similar.html]

motif24

## Information for motif24

T
G
A
C
C
T
A
G
A
T
C
G
A
T
G
C
G
A
T
C
A
G
T
C
A
G
T
C
C
G
A
T
A
T
G
C
A
G
T
C
G
A
T
C
G
A
T
C
G
T
A
C
  
Reverse Opposite:  

C
A
T
G
C
T
A
G
C
T
A
G
A
C
T
G
A
T
C
G
G
C
T
A
C
T
A
G
C
T
A
G
C
T
A
G
T
A
C
G
T
A
G
C
G
A
T
C
A
C
T
G
  

|  |  |
| --- | --- |
| p-value: | 1e-45 |
| log p-value: | -1.041e+02 |
| Information Content per bp: | 1.749 |
| Number of Target Sequences with motif | 2005.0 |
| Percentage of Target Sequences with motif | 15.19% |
| Number of Background Sequences with motif | 3814.1 |
| Percentage of Background Sequences with motif | 10.45% |
| Average Position of motif in Targets | 550.1 +/- 553.5bp |
| Average Position of motif in Background | 324.7 +/- 191.5bp |
| Strand Bias (log2 ratio + to - strand density) | 0.2 |
| Multiplicity (# of sites on avg that occur together) | 1.46 |
| Motif File: | file (matrix) reverse opposite |

### Similar de novo motifs found

|  |  |  |  |  |  |  |  |
| --- | --- | --- | --- | --- | --- | --- | --- |
| Rank | Match Score | Redundant Motif | P-value | log P-value | % of Targets | % of Background | Motif file |
| 1 | 0.837 | A C T G A T G C G A T C T G A C A G T C A C T G A T G C T A G C A G T C | 1e-43 | -100.916676 | 9.93% | 6.16% | motif file (matrix) |
| 2 | 0.963 | A T C G T A C G A T G C G A T C G A T C A G T C G A C T A T G C A G T C G A T C G T A C A T G C | 1e-43 | -100.150542 | 13.72% | 9.28% | motif file (matrix) |
| 3 | 0.849 | T C A G T A C G A T G C G A T C G A T C G A T C A C T G A T G C A T G C G A T C | 1e-42 | -97.377391 | 11.93% | 7.85% | motif file (matrix) |
| 4 | 0.795 | A C T G A T C G A T C G T C A G T C A G T C A G A T C G A C T G | 1e-41 | -95.570366 | 22.32% | 16.90% | motif file (matrix) |
| 5 | 0.822 | C T A G A C T G T A C G A T G C C T A G C T A G T C A G A T C G T A G C A T G C T C A G | 1e-40 | -94.394305 | 9.87% | 6.22% | motif file (matrix) |
| 6 | 0.859 | C T A G T C A G T A C G C G T A C A T G C T A G C T A G T A C G A T G C G T A C G A T C | 1e-39 | -91.471246 | 16.47% | 11.84% | motif file (matrix) |
| 7 | 0.761 | A T G C G A C T A G T C A G T C G T A C A G T C A G T C T A G C G A T C | 1e-27 | -64.103424 | 19.97% | 15.72% | motif file (matrix) |
| 8 | 0.843 | C A T G A T C G A C T G C T A G C T A G C T A G C T A G T C A G C T A G A C T G | 1e-27 | -63.539941 | 19.03% | 14.88% | motif file (matrix) |
| 9 | 0.787 | A T C G A T C G A T C G C G T A A C T G A T C G A C T G | 1e-26 | -61.076989 | 26.69% | 22.03% | motif file (matrix) |
| 10 | 0.842 | T C A G A C T G A G T C A G T C A G T C A G T C C T A G A T C G A G T C A G T C A G T C A G T C C G T A A T C G | 1e-24 | -55.812660 | 13.77% | 10.41% | motif file (matrix) |
